# A Well-Deserved Recognition

**Published:** 1900-01

**Authors:** 


### A WELL-DESERVED RECOGNITION.

THE profession over the entire country will with one accord approve heartily the action of Governor Stone, of Pennsylvania, in the reappointment of Dr. Leonard Pearson, State Veterinarian. The overwhelming testimony as to the valued services of Dr. Pearson to the people of Pennsylvania in placing veterinary sanitary work on a strong basis, on conservative plans that have now the support of the live-stock owners of the whole Commonwealth, not to speak of the monetary losses conserved, the more valued health of the people preserved, and the reduction of losses that fell heavily on an already burdened class of our people, met a hearty response at the hands of the Governor, and this new lease of power is sure to add again to the interests of our people and be more and more appreciated by the entire country who are watching zealously the outcome of the plans in Pennsylvania dealing with State problems of the greatest moment.

